# Having older siblings is associated with gut microbiota development during early childhood

**DOI:** 10.1186/s12866-015-0477-6

**Published:** 2015-08-01

**Authors:** Martin Frederik Laursen, Gitte Zachariassen, Martin Iain Bahl, Anders Bergström, Arne Høst, Kim F. Michaelsen, Tine Rask Licht

**Affiliations:** Division of Diet, Disease Prevention and Toxicology, National Food Institute, Technical University of Denmark, Mørkhøj Bygade 19, DK-2860 Søborg, Denmark; H.C. Andersen Children’s Hospital, Odense University Hospital, Sdr. Boulevard 29, DK-5000 Odense C, Denmark; Department of Nutrition, Exercise and Sports, University of Copenhagen, Rolighedsvej 30, DK-1958 Frederiksberg C, Denmark

**Keywords:** Alpha diversity, Asthmatic bronchitis, Early life infections, Eczema furred pets, Infants, Gut microbiota, Older siblings, Richness, SKOT I cohort

## Abstract

**Background:**

Evidence suggests that early life infections, presence of older siblings and furred pets in the household affect the risk of developing allergic diseases through altered microbial exposure. Recently, low gut microbial diversity during infancy has also been linked with later development of allergies. We investigated whether presence of older siblings, furred pets and early life infections affected gut microbial communities at 9 and 18 months of age and whether these differences were associated with the cumulative prevalence of atopic symptoms of eczema and asthmatic bronchitis at 3 years of age. Bacterial compositions and diversity indices were determined in fecal samples collected from 114 infants in the SKOT I cohort at age 9 and 18 months by 16S rRNA gene sequencing. These were compared to the presence of older siblings, furred pets and early life infections and the cumulative prevalence of diagnosed asthmatic bronchitis and self-reported eczema at 3 years of age.

**Results:**

The number of older siblings correlated positively with bacterial diversity (*p* = 0.030), diversity of the phyla *Firmicutes* (*p* = 0.013) and *Bacteroidetes* (*p* = 0.004) and bacterial richness (*p* = 0.006) at 18 months. Further, having older siblings was associated with increased relative abundance of several bacterial taxa at both 9 and 18 months of age. Compared to the effect of having siblings, presence of household furred pets and early life infections had less pronounced effects on the gut microbiota. Gut microbiota characteristics were not significantly associated with cumulative occurrence of eczema and asthmatic bronchitis during the first 3 years of life.

**Conclusions:**

Presence of older siblings is associated with increased gut microbial diversity and richness during early childhood, which could contribute to the substantiation of the hygiene hypothesis. However, no associations were found between gut microbiota and atopic symptoms of eczema and asthmatic bronchitis during early childhood and thus further studies are required to elucidate whether sibling-associated gut microbial changes influence development of allergies later in childhood.

**Electronic supplementary material:**

The online version of this article (doi:10.1186/s12866-015-0477-6) contains supplementary material, which is available to authorized users.

## Background

More than two decades ago, David Strachan proposed that the inverse relationship observed between household size and prevalence of hay fever and eczema *could be explained if allergic diseases were prevented by infection in early childhood*, *transmitted by unhygienic contact with older siblings* [1]. This would later become known as the *hygiene hypothesis*. Since then, numerous epidemiological studies have confirmed his results, as reviewed by Karmaus and Botezan [2]. A recent comprehensive worldwide study from the International Study of Asthma and Allergies in Children (ISAAC) concluded that eczema and hay fever are indeed inversely associated with number of older siblings, and that this association is mainly seen in affluent countries [3]. Additional environmental factors affecting the risk of allergies have been identified, such as furred pets, which are associated with decreased risk [4, 5] and birth by cesarean section [6] and use of antibiotics [7], which are both associated with increased risk of allergies. Obviously, these associations are not solely due to transmission of infectious microbes, but rather reflect a generally altered exposure to non-pathogenic microbes [8]. This suggests that not only early life infections, but many aspects of altered microbial exposure are affecting development of allergies. Specifically, the important role of the endogenous gut microbiota and its interaction with the immune system has become evident [9]. In particular a reduced diversity in the early gut microbiota has recently been linked with development of both eczema and asthma [10–14]. However, very limited research has presently been done to address the impact of early life infections, older siblings and furred pets on development of the infant gut microbiota, and thus it is still largely unknown how these are linked to development of allergies. Therefore, we aimed to investigate associations between (i) environmental factors, including older siblings, furred household pets and early life infections, (ii) infant microbial gut communities at ages 9 and 18 months and (iii) the cumulative prevalence of atopic symptoms of eczema and asthmatic bronchitis at the age of 3 years in 114 Danish children within the SKOT I cohort [15].

## Methods

### The SKOT I cohort

The present study is based on the SKOT I cohort, which includes 311 demographically similar Danish children followed during the first 3 years of life with the overall aim to elucidate relationships between early diet, growth and development and later disease risks [15–17]. Background characteristics of the study population has been published previously [16, 18] and we have previously described the gut bacterial population of this cohort [17], however with a less comprehensive methodology than applied here. The study protocol was approved by the Committees on Biomedical Research Ethics for the Capital Region of Denmark (H-KF-2007-0003) and written consent was given by parents. Inclusion criteria for the SKOT I cohort were single birth and full term delivery, absence of chronic illness and age of 9 months ± 2 weeks at start of study. The participants in the SKOT I cohort were recruited by sending an invitation to a random sample of 2211 families [15]. Fifteen percent responded (330), 19 dropped out before first examination and thus 311 were included in the cohort. 290 of the infants were seen both at the 9 and 18 month visit. After exclusion of individuals with fecal samples taken at only one time point, use of antibiotics and inappropriately stored fecal samples (227), a randomly selected subset of 114 samples was used in the present study. Fecal samples obtained at 9 and 18 months of age were freshly delivered on the morning of visitation or had been stored in the participant’s home, in provided freezer containers, either in the freezer (−18 °C) or in the fridge (4 °C) for maximally 24 h before delivery to University of Copenhagen, Department of Nutrition, Exercise and Sports, where they were stored at −80 °C until DNA extraction. Information about household older siblings (categorized into 0, 1 or >2, due to very few infants having more than 2 older siblings) and furred pets (cats, dogs or rabbits in the household during the first 18 months of life) was collected at parental interviews, which were performed at the university and carried out by a trained staff at 9 and 18 months of age, respectively (Additional file 1: Table S1). Early life infections (defined as parentally reported recurrent otitis media or pneumonia initiated before the age of 12 months), allergic heredity (parents and/or siblings diagnosed with food allergy, eczema, hay fever, urticaria or asthma) and cumulative prevalence (0–3 years of age) of diagnosed asthmatic bronchitis and self-reported eczema were collected at parental interviews at 3 years of age (Additional file 1: Table S1). Possible confounding factors on gut microbiota including mode of delivery, gestational age at birth, infant age at 9 and 18 month examinations, age at start of daycare/nursery, breast feeding duration and daily macronutrient intake at 9 and 18 months of age (Additional file 2: Table S2) were used to evaluate possible differences between infants with or without older siblings, furred pets and early life infections.

### DNA extraction and PCR amplification of the 16S rRNA gene

DNA was extracted (PowerLyzer® PowerSoil® DNA isolation kit, MoBio 12855–100) from 250 mg feces according to the provided protocol with minor modifications: bead beating was performed at 30 cycles/s for 10 min (Retsch MM 300 mixer mill) and the initial centrifugation steps were performed at 10,000 × g for 3 min, as recommended for clay matter. DNA quantity and quality were measured by Qubit® dsDNA BR assay (Invitrogen™, Q32850) and NanoDrop® 1000 (Thermo Scientific), respectively, yielding 33.3 ± 22.2 ng/μl DNA with A260/A280 = 1.82 ± 0.12 and A260/A230 = 1.61 ± 0.38. The PCR amplification of the V3-region of the 16S rRNA gene was performed with 5 ng community DNA as template, using 0.2 μl Phusion High-Fidelity DNA polymerase (Fisher Scientific, F-553 L), 4 μl HF-buffer, 0.4 μl dNTP (10 mM of each base), 1 μM forward primer (PBU 5′-A-adapter-TCAG-barcode-CCTACGGGAGGCAGCAG-3′) and 1 μM reverse primer (PBR 5′-trP1-adapter-ATTACCGCGGCTGCTGG-3′) in a 20 μl total reaction volume. Both primers included sequencing adaptors and the forward primer additionally a unique 10–12 bp barcode (Ion Xpress™ Barcode Adapters). The PCR program included 30s at 98 °C, 24 cycles of 15 s at 98 °C and 30 s at 72 °C, followed by 5 min at 72 °C. The PCR product was purified using HighPrep™ PCR Magnetic Beads (MAGBIO®, AC-60005) with the 96-well magnet stand (MAGBIO®, MyMag 96), according to the prescribed procedure. DNA quantity was measured using Qubit® dsDNA HS assay (Invitrogen™, Q32851) and samples were pooled to obtain equimolar libraries containing up to 90 samples in each library.

### Sequencing and data handling

Sequencing of the 16S rRNA gene libraries was performed using the Ion OneTouch™ and Ion PGM systems with a 318-Chip, generating 5–7 million reads per chip with a median length of 180 bp. Sequencing data were imported into CLC Genomic Workbench (version 7.0.3, CLC bio, Qiagen, Aarhus, DK), reads were demultiplexed and trimmed to remove low quality sequences (*p*_*base*-*calling error*_ = 0.05), ambiguous nucleotides (maximally 2 allowed), primers and barcodes and to discard reads below 110 bp and above 180 bp. Sequencing data is deposited at NCBI Sequence Read Archive with the Accession Number SRP052851, under the BioProject Accession Number PRJNA273694. The sorted and trimmed FASTA files were run through the RDP classifier [19] with a bootstrap cutoff of 50 % as recommended for sequences shorter than 250 bp [20]. The total number of reads for each sample was 46418 ± 17806 and was used to calculate the relative abundance of each bacterial group. In the further analysis a cutoff of 0.01 % in mean relative abundance at either 9 or 18 months was applied. Based on the detection limit (1 read), a threshold was set to 0.001 % (~0.46 reads) and zeroes were assigned this value.

### Data analysis and statistical tests

Alpha diversity (Shannon index) was calculated at ages 9 and 18 months based on relative abundance of all identified genera (bacterial diversity) or all identified genera within the four major infant gut associated phyla of *Firmicutes*, *Bacteroidetes*, *Actinobacteria* and *Proteobacteria* (phylum diversity) in each sample, using R (version 3.1.0, R Core Team 2014. R: A language and environment for statistical computing. R Foundation for Statistical Computing, Vienna, Austria. URL: http://www.R-project.org/) package *vegan* [21]. Richness was assessed by randomly subsampling 8000 reads per sample and calculating the average number of observed genera within each sample using the R package *vegan*. Statistical tests were performed in GraphPad Prism (version 5.0.3, GraphPad Software Inc., La Jolla, CA). Non-parametric Mann-Whitney tests were used to test for differences in medians of alpha diversity and richness between children with and without older siblings, furred pets and early life infections and between children with or without asthmatic bronchitis or eczema during the first 3 years of life. Spearman correlation analyses were performed to address associations between older siblings (0, 1 or >2), furred pets, early life infections and the relative abundance of gut bacterial genera at 9 and 18 months of age, which were also correlated with the binary variables asthmatic bronchitis and eczema during the first 3 years of life. Correlation matrices were illustrated using the R package *corrplot* [22]. *P*-values were adjusted for multiple testing using a false discovery rate of 10 % [23]. Using the R package *FactoMineR* [24] principal components were calculated from data on relative abundance of all bacterial families.

## Results

### Effect of older siblings, furred pets and early life infections on gut microbiota

**Fig. 1:**
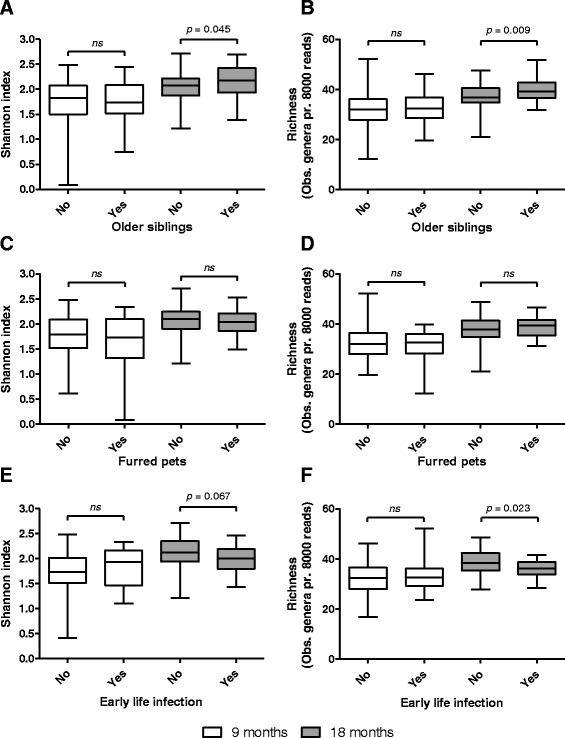
Boxplots comparing bacterial diversity and richness at 9 months (white color) and 18 months (grey color) between individuals with or without older siblings (**a**, **b**), furred pets (**c**, **d**) and early life infections (**e**, **f**). Boxes show 25^th^ to 75^th^ percentiles and whiskers indicate minimum to maximum values. Statistical significance was evaluated by Mann Whitney test, using *p* < 0.05 as measure of significance. *ns* = not significant, however *p*-values below 0.1 are shown

**Fig. 2:**
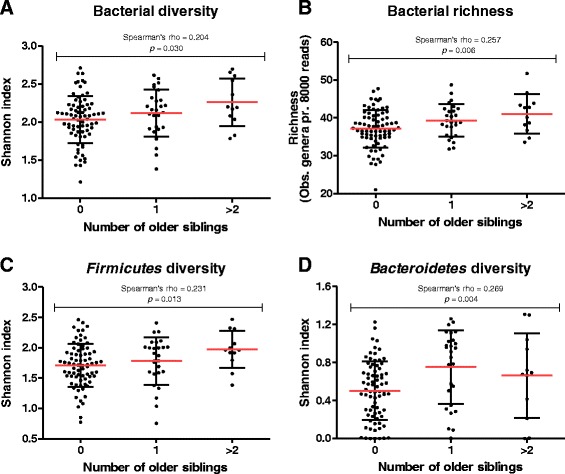
Scatter plots illustrating the bacterial diversity (**a**), richness (**b**) and phyla diversities of *Firmicutes* (**c**) and *Bacteroidetes* (**d**) in infants at 18 months of age divided into groups according to the number of older siblings in the household. Red lines indicate mean ± standard deviation. Spearman’s correlations coefficient rho and *p*-value (*p* < 0.05 as a measure of significance) are shown

**Fig. 3:**
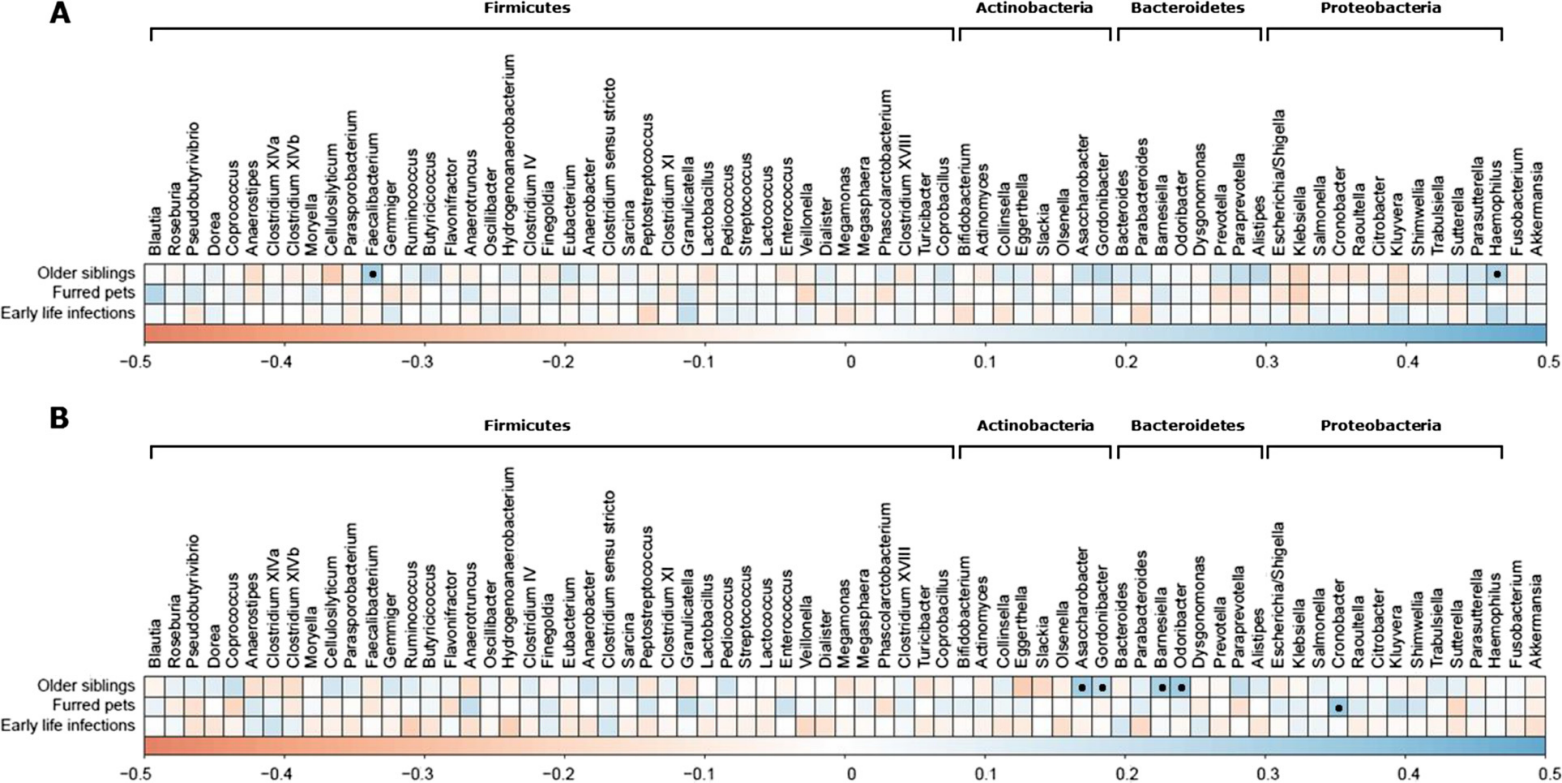
Correlation matrices relating relative abundance of bacterial genera at 9 months (**a**) and 18 months (**b**) to the presence of older siblings, furred pets and early life infections. Scale indicate the Spearman’s rank correlation coefficient rho, ranging from −0.5 (negative correlation; red color) to 0.5 (positive correlation; blue color). Black dots indicate statistically significant correlations with FDR adjusted *p*-value < 0.1

To minimize confounding effects, we confirmed that prevalence of allergic heredity and C-section, average gestational age at birth, actual age at 9 and 18 month visits, infant age at start of daycare or nursery, breastfeeding duration and macronutrient intake at 9 and 18 months visits were similar between infants with and without older siblings, furred pets or early life infections (Additional file 2: Table S2). Alpha diversity and richness of the gut microbial populations were calculated for all individuals at ages 9 and 18 months, and these data were compared between infants with and without older siblings, furred pets and early life infections (Fig. 1). Infants with older siblings in the household had a higher bacterial diversity (*p* = 0.045) and richness (*p* = 0.009) at 18, but not at 9 months of age compared to infants with no older siblings (Fig. 1a, b). In addition, significant correlations between the specific number of older siblings (0, 1 or >2) and bacterial diversity (*p* = 0.030) or richness (*p* = 0.006) were found (Fig. 2a, b). Specifically, the phyla diversity within *Firmicutes* (*p* = 0.013) and *Bacteroidetes* at age 18 months (*p* = 0.004) were positively correlated with numbers of older siblings (Fig. 2c, d), while phyla diversity within *Actinobacteria* and *Proteobacteria* were not affected (data not shown). Presence of furred pets in the household did not affect bacterial diversity or richness of the total gut microbial populations at 9 or 18 months (Fig. 1c, d). However, infants with furred pets had lower diversity (*p* = 0.010) within the *Firmicutes* phylum at 9 months (data not shown). Infants with registered history of early life infections had a lower bacterial diversity (*p* = 0.067) and richness (*p* = 0.023) at age 18 months, but not at age 9 months (Fig. 1e, f). Investigation of associations between abundances of specific microbial genera and the presence of older siblings, furred pets or early life infections (Fig. 3) revealed that *Haemophilus* and *Faecalibacterium* abundance at age 9 months were significantly positively associated with the presence of older siblings, while this was true for *Barnesiella*, *Odoribacter*, *Asaccharobacter* and *Gondonibacter* at age 18 months. The presence of furred pets was positively associated only with *Cronobacter* abundance at 18 months of age. Early life infections were not significantly associated with any specific gut microbial genera after adjustment for multiple testing. However, we note that *Haemophilus* abundance at 9 months was positively associated (non-adjusted *p* = 0.019) with early life infections (Additional file 3: Figure S1).

### Effect of gut microbiota on cumulative prevalence of eczema and asthmatic bronchitis

**Fig. 4:**
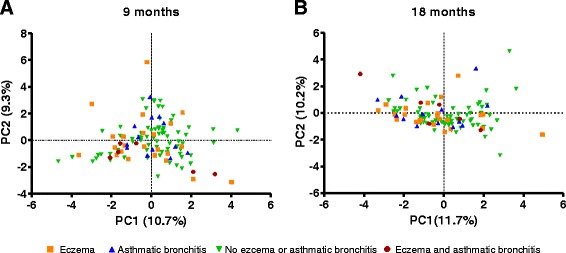
Principle Component Analysis plots of relative abundance of gut bacterial families at 9 (**a**) and 18 months of age (**b**). Green triangles indicate no eczema or asthmatic bronchitis, orange squares indicate presence of eczema, blue triangles indicate presence of asthmatic bronchitis and red circles indicate presence of both asthmatic bronchitis and eczema

Principal component analysis of the gut microbiota composition at family level at ages 9 and 18 months did not reveal any separation of samples originating from children with symptoms of eczema, asthmatic bronchitis or both, compared to samples originating from children without these symptoms (Fig. 4). In accordance with this, Spearman correlation analysis of relative abundances of bacterial genera at 9 and 18 months of age against the occurrence of asthmatic bronchitis and eczema during the first 3 years of life revealed no significant correlations with these outcomes after correction for multiple testing (Additional file 4: Figure S2). Neither asthmatic bronchitis nor eczema was found to be associated with diversity or richness of the faecal microbial populations (Additional file 5: Figure S3).

## Discussion

We recently, using less comprehensive methodology, profiled the gut microbiota development of the cohort analysed in the present study [17], yet the possible associations with atopic symptoms and presence of early life infections, furred pets and older siblings in the household were not previously analysed. When assessing the effects of these external factors on infant gut microbiota, gestational age at birth [25], mode of delivery [26], infant age at sampling [27], start of daycare or nursery, breast feeding [17], dietary patterns [28] and use of antibiotics [29] are among the most likely possible confounding factors. We therefore excluded infants with current use of antibiotics and confirmed that C-section prevalence, average gestational age at birth, actual infant age at 9 and 18 month visits, infant age at start of daycare or nursery, breast feeding duration and nutrient intake at 9 or 18 months of age between infants with and without older siblings, furred pets and early life infections were similar (Additional file 2: Table S2). Judged by the high similarity between these groups, these factors seem unlikely to confound our results. However, we cannot exclude the possibility that other factors might affect our results.

In general, it is a limitation of our study that a relatively low number of infants had early life infections and furred pets, which might explain that only few associations were observed. Nonetheless, in children with registered early life infections, diversity and richness measures were lower at 18 months, but not at 9 months than in children with no early life infections. For most subjects’ recurrence of infections were present between 9 and 18 months (Additional file 1: Table S1). Thus, even though we excluded individuals with current antibiotics use at time of sampling and recorded no use of antibiotics 7-days prior to 18 month visits, earlier use in connection with an infection might still impact diversity/richness at the sampling point of 18 months. In agreement with this, antibiotic consumption is reported to reduce bacterial alpha diversity as well as richness, and the effects may be present for a long time after the treatment has ended [30, 31]. Presumably, potential beneficial effects of early life infections on the development of allergies [32] might be counteracted by treatment of these infections with antibiotics [7].

Infants with furred pets had a lower *Firmicutes* diversity at 9 months of age and higher abundance of *Cronobacter* at 18 months of age. Very few studies have included investigations of the effects of furred pets on gut microbiota composition [33–35]. While a study in 24 Canadian infants at 4 months of age found over-representation of *Clostridiaceae*, *Veillonella*, *Peptostreptococcaceae* and *Coprococcus* and under-representation of *Bifidobacteriaceae* in infants living with pets [34], two studies in much larger cohorts found no effect of having pets in the household at 1 and 6 months on the gut microbiota composition [33, 35]. Obviously, while differences in pet-types and exposure time could explain these discrepancies, better controlled studies are required to assess the impact of different pets on gut microbiota.

The presence of older siblings in the household significantly affected the infant gut microbial community. Bacterial diversity and richness were significantly higher at 18 months, but not at 9 months for infants having one or more older siblings, compared to infants without older siblings. The absence of a sibling-effect on diversity/richness at 9 months is in agreement with a previous study reporting no significant differences in Shannon index and Chao1 richness estimate between infants with and without older siblings at age 4 months [34]. Also the specific number of older siblings was positively associated with bacterial alpha diversity and richness as well as diversity within the *Bacteroidetes* and *Firmicutes* phyla that are both typical in an adult-like microbiota composition. We suggest that the effect of siblings on gut microbial diversity/richness increases with time during the first years of life, due to increased contact with older siblings. Additionally, it is likely that parental behaviour, such as hygiene practices, is altered as more children are born in the family. We find it plausible that presence of older siblings in the household is more likely to affect the number of different bacteria to which an infant is exposed, affecting richness and diversity, than to affect the relative abundances of specific bacterial taxa in the gut. However, we did observe that the relative abundance of a few genera, namely *Faecalibacterium* and *Haemophilus* at 9 months was higher in infants with older siblings. *Faecalibacterium prausnitzii* is one of the most abundant species within the gastrointestinal tract of adult humans and its abundance rises quickly during the first years of life [17]. It contributes to butyrate production by degradation of non-digestible dietary fibres like pectin and inulin [36], and has been reported to elicit anti-inflammatory effects *in vitro* [37]. *Haemophilus* species colonize the nasopharynx and upper respiratory tract in approximately 60 % of non-symptomatic children [38]. Thus, older siblings might transfer these microbes through close contact. A particularly interesting notion though, was that *Haemophilus* levels at 9 months were also positively associated with early life infections (otitis media or pneumonia). *Haemophilus influenzae* is known to be involved in both pneumonia and otitis media [39, 40] and is suggested to be transferred from older siblings with these infections [41] or by other children at day-care facilities [38], which might explain our observations. At age 18 months, strict anaerobic genera within the *Bacteroidetes* phylum (*Odoribacter* and *Barnesiella*) were positively correlated with the presence of older siblings. This is consistent with the increased diversity of *Bacteroidetes* observed in infants with older siblings. In support of this, a previous study showed that the ratio of strict to facultative anaerobes 12 months after birth was lower in infants without older siblings than infants with older siblings [33], suggesting that infants acquire strict anaerobic bacteria from their older siblings. Only few other studies have addressed the effect of older siblings on specific gut microbes. These studies have reported that having older siblings is associated with increased colonization rates of *Lactobacillus* and *Bacteroides* and decreased colonization rates of *Clostridium* at age 5 weeks [42], lower abundance of *Peptostreptococcaceae* at age 4 months [34], as well as higher abundance of *Bifidobacterium* and lower abundance of *Enterobacteriaceae* during infancy [43]. While we could not confirm these results, difference in age, geography, culture, eating habits and other confounding factors or different methodological procedures could explain that studies report different outcomes with respect to effects on specific bacterial taxa.

Several studies have reported a link between reduced gut microbial diversity in infancy and later development of eczema [10, 11, 13, 14] and asthma [12], whereas another large study involving a high risk population, found no associations between diversity and eczema or asthma [44]. With the given power, we found no association between gut microbial composition and diversity at 9 or 18 months and occurrence of atopic symptoms of eczema or asthmatic bronchitis assessed during the first 3 years of life. However, excluding infants with atopic symptoms of eczema before the age of 18 months (*n* = 10), we saw a tendency (*p* = 0.079) for reduced bacterial diversity in infants with atopic symptoms eczema occurring after the age of 18 months (*n* = 19). This could be relevant since the effect of older siblings on bacterial diversity seems to be evident at 18 months, but not 9 months of age. Most previous studies reporting such an association measured gut microbial diversity in early infancy [10–14, 44], typically the first weeks or months of life, where the gut microbiota is more unstable than in late infancy/early childhood [45, 46], possibly explaining the differences in results. Only one previous study included bacterial diversity at 18 months of age and reported a higher diversity in individuals with eczema compared to healthy controls [47]. However, this was based on only 24 individuals and dietary patterns at 18 months were not recorded, despite that diet is one of the strongest factors affecting gut microbiota [48] and thus a potential confounder. Differences in methods of assessing atopic symptoms could also contribute to inconsistencies between results. The fact that eczema was parentally self-reported is a limitation of our study, since a number of other skin symptoms might be interpreted as eczema [49]. Furthermore, asthmatic bronchitis is often caused by a viral infection in early childhood and might not be indicative of actual asthma development [50]. Recall bias is another issue related to questionnaires assessing symptoms of atopy. Therefore, inadequate assessment of what are actual symptoms of allergy might explain discrepancies compared to previous studies reporting a link between reduced gut microbial diversity and allergy development. In light of the recent strong epidemiological evidence provided by ISAAC [3] of a sibling effect on prevalence of eczema in children at both 6–7 years and 13–14 years of age, we suspect that the increased gut microbial diversity/richness in early childhood, associated with the number of older siblings, could contribute to the lower eczema prevalence observed later in childhood.

## Conclusion

We found that the presence of older siblings in the household significantly associates with increased gut microbial diversity and richness during early childhood. This has to our knowledge not previously been shown and could contribute to the substantiation of the hygiene hypothesis. However, gut microbiota during early childhood was not associated with cumulative occurrence of atopic symptoms of eczema or asthmatic bronchitis at 3 years of age. Further studies are warranted to elucidate the possible implications of sibling-associated gut microbial changes on development of allergies in later childhood.

## Additional files

Additional file 1: Table S1. Number of children with early life infections, older siblings, furred pets, family history of allergy and cumulative prevalence of reported eczema and asthmatic bronchitis assessed at 3 years of age. Additional file 2: Table S2. Possible confounding factors for effects of older siblings, furred pets and early life infections on gut microbiota: Family history of allergy, C-section, gestational age at birth, infant age at 9 and 18 month visits, infant age at start of daycare/nursery, duration of breastfeeding and daily nutrient intake at 9 and 18 months of age for infants with and without older siblings, furred pets and early life infections. Additional file 3: Figure S1. Relative abundance of *Haemophilus* at 9 and 18 months of age in individuals with or without early life infections (A) and older siblings (B). Additional file 4: Figure S2. Correlation matrices relating relative abundance of gut bacterial genera at 9 months (A) and 18 months (B) to the presence of asthmatic bronchitis and eczema. Additional file 5: Figure S3. Boxplots comparing bacterial diversity and richness at 9 and 18 months between individuals with or without eczema (A-B) and asthmatic bronchitis (C-D).

## Abbreviations

SKOT I: a Danish abbreviation for “Dietary habits and wellbeing of young children”
