# Supplementary material for: Having older siblings is associated with gut microbiota development during early childhood
Source: BMC Microbiol. 2015 Aug 1;15:154. doi: 10.1186/s12866-015-0477-6 (PMC4522135; doi:10.1186/s12866-015-0477-6)
Supplement: Additional file 1: Table S1. — Number of children with early life infections, older siblings, furred pets, family history of allergy and cumulative prevalence of reported eczema and asthmatic bronchitis assessed at 3 years of age. [file 12866_2015_477_MOESM1_ESM.docx]

Table S1 - Number of children with early life infections, older siblings, furred pets, family history of allergy and cumulative prevalence of eczema and asthmatic bronchitis assessed at three years of age.

|  | | | **Number of children^#^** | **Period of reoccurring symptoms** | | |
| --- | --- | --- | --- | --- | --- | --- |
|  |  |  |  | **Onset month ± sd** | **Offset month ± sd** | **Range (months)** |
| ***Early life infections*** | | | **95** | **-** | **-** | **-** |
|  | *No* | | 75 | **-** | **-** | **-** |
|  | *Yes* | | 20 | 9.0 ± 2.0 | 22.6 ± 6.2 | 5-36+ |
|  |  | *Otitis media* | 17 | 8.8 ± 2.1 | 22.2 ± 6.2 | 5-36+ |
|  |  | *Pneumonia* | 4 | 9.8 ± 1.7 | 24.0 ± 6.9 | 8-30 |
| ***Older siblings*** | | | **114** | **-** | **-** | **-** |
|  | *No* | | 75 | **-** | **-** | **-** |
|  | *Yes* | | 39 | **-** | **-** | **-** |
|  |  | *1* | 27 | **-** | **-** | **-** |
|  |  | *2* | 9 | **-** | **-** | **-** |
|  |  | *3* | 1 | **-** | **-** | **-** |
|  |  | *4* | 2 | **-** | **-** | **-** |
| ***Furred pets*** | | | **107** | **-** | **-** | **-** |
|  | *No* | | 86 | **-** | **-** | **-** |
|  | *Yes* | | 21 | **-** | **-** | **-** |
|  |  | *Cats* | 14 | **-** | **-** | **-** |
|  |  | *Dogs* | 4 | **-** | **-** | **-** |
|  |  | *Rabbits* | 3 | **-** | **-** | **-** |
| ***Eczema^1^*** | | | **102** | **-** | **-** | **-** |
|  | *No* | | 73 | **-** | **-** | **-** |
|  | *Yes* | | 29 | 12.9 ± 11.2 | 28.5 ± 11.4 | 0-36+ |
| ***Asthmatic*** ***bronchitis^2^*** | | | **104** | **-** | **-** | **-** |
|  | *No* | | 85 | **-** | **-** | **-** |
|  | *Yes* | | 19 | 16.8 ± 8.3 | 25.6 ± 7.9 | 7-36+ |
| ***Family*** ***history*** ***of*** ***allergy^3^*** | | | **104** | **-** | **-** | **-** |
|  | *No* | | 42 | **-** | **-** | **-** |
|  | *Yes* | | 62 | **-** | **-** | **-** |

#) Not in all cases data was available for all 114 infants.

1) Itching, red and dry rash, sometimes with watery small blisters. Affecting the same skin areas recurrently, often elbow joint, knee joint and neck.

2) Squeaky and wheezing breathing in connection with cold or other viral infections in the respiratory system.

3) Parents and/or siblings previously diagnosed with food allergy, eczema, hay fever, urticaria or asthma.
