# Supplementary material for: Having older siblings is associated with gut microbiota development during early childhood
Source: BMC Microbiol. 2015 Aug 1;15:154. doi: 10.1186/s12866-015-0477-6 (PMC4522135; doi:10.1186/s12866-015-0477-6)
Supplement: Additional file 2: Table S2. — Possible confounding factors for effects of older siblings, furred pets and early life infections on gut microbiota: Family history of allergy, C-section, gestational age at birth, infant age at 9 and 18 month visits, infant age at start of daycare/nursery, duration of breastfeeding and daily nutrient intake at 9 and 18 months of age for infants with and without older siblings, furred pets and early life infections. [file 12866_2015_477_MOESM2_ESM.docx]

Table S2 – Possible confounding factors for effects of older siblings, furred pets and early life infections on gut microbiota: Family history of allergy, C-section, gestational age at birth, infant age at 9 and 18 month visits, infant age at start of daycare/nursery, duration of breastfeeding and daily nutrient intake at 9 and 18 months of age for infants with and without older siblings, furred pets and early life infections.

| **Possible confounding factor** | | ***Older siblings*** | | ***Furred pets*** | | ***Early life infections*** | |
| --- | --- | --- | --- | --- | --- | --- | --- |
|  |  | **No (n=75)** | **Yes (n=39)** | **No (n=86)** | **Yes (n=21)** | **No (n=75)** | **Yes (n=20)** |
| **Family history of allergy** | |  |  |  |  |  |  |
|  | Prevalence of allergic heredity (%) | 63.2 | 52.8 | 62.2 | 47.4 | 56.8 | 65.0 |
| **Birth** | |  |  |  |  |  |  |
|  | C-section (%) | 13.3 | 12.8 | 14.0 | 9.5 | 12.0 | 21.1 |
|  | Gestational age at birth (weeks±sd) | 40.2±1.1 | 40.1±1.2 | 40.2±1.2 | 40.0±1.2 | 40.1±1.2 | 40.1±1.1 |
| **Infant age at** | |  |  |  |  |  |  |
|  | 9 month visit (months±sd) | 9.1±0.3 | 9.1±0.3 | 9.1±0.3 | 9.0±0.3 | 9.1±0.3 | 9.1±0.3 |
|  | 18 month visit (months±sd) | 17.9±0.5 | 17.9±0.5 | 17.9±0.5 | 18.0±0.5 | 17.9±0.5 | 18.1±0.5 |
|  | Start of daycare or nursery (months±sd) | 11.3±2.1 | 11.8±2.9 | 11.6±2.3 | 11.4±2.3 | 11.4±2.3 | 11.7±2.1 |
| **Breastfeeding duration** | |  |  |  |  |  |  |
|  | Exclusively breastfed (days±sd) | 106.7±55.7 | 115.3±54.2 | 109.2±58.4 | 110.2±49.2 | 108.5±54.7 | 107.1±59.1 |
|  | Breastfed (days±sd) | 248.9±116.5 | 253.8±106.8 | 246.3±108.0 | 265.9±128.2 | 241.1±111.4 | 241.0±104.7 |
| **Nutrient intake 9 months of age** | |  |  |  |  |  |  |
|  | Energy intake (kJ/day/kg±sd) | 382.7±104.3 | 368.7±120.0 | 382.9±112.8 | 379.5±84.3 | 375.6±117.1 | 410.3±90.8 |
|  | Protein intake (g/day/kg±sd) | 2.7±0.9 | 2.7±0.9 | 2.8±0.9 | 2.7±0.7 | 2.7±0.9 | 3.0±0.8 |
|  | Fat intake (g/day/kg±sd) | 3.7±1.2 | 3.5±1.3 | 3.7±1.3 | 3.7±0.9 | 3.6±1.3 | 3.9±1.2 |
|  | Carbohydrate intake (g/day/kg±sd) | 11.5±3.3 | 11.2±3.8 | 11.6±3.5 | 11.5±2.8 | 11.3±3.7 | 12.5±2.8 |
|  | Fibre intake (g/day/kg±sd) | 1.0±0.4 | 0.9±0.4 | 1.0±0.4 | 0.9±0.3 | 0.9±0.4 | 1.1±0.3 |
| **Nutrient intake 18 months of age** | |  |  |  |  |  |  |
|  | Energy intake (kJ/day/kg±sd) | 426.8±98.1 | 411.5±92.7 | 415.8±97.3 | 432.6±87.0 | 421.1±81.6 | 406.8±116.7 |
|  | Protein intake (g/day/kg±sd) | 3.7±0.9 | 3.6±0.9 | 3.6±0.9 | 3.8±1.1 | 3.6±0.8 | 3.6±1.1 |
|  | Fat intake (g/day/kg±sd) | 3.9±1.2 | 3.7±1.1 | 3.8±1.2 | 3.7±0.9 | 3.8±1.0 | 3.7±1.3 |
|  | Carbohydrate intake (g/day/kg±sd) | 12.7±2.9 | 12.5±2.7 | 12.3±2.8 | 13.4±2.8 | 12.7±2.4 | 12.0±3.2 |
|  | Fibre intake (g/day/kg±sd) | 1.1±0.3 | 1.0±0.3 | 1.0±0.3 | 1.1±0.4 | 1.0±0.3 | 1.0±0.3 |
