# Supplementary material for: Having older siblings is associated with gut microbiota development during early childhood
Source: BMC Microbiol. 2015 Aug 1;15:154. doi: 10.1186/s12866-015-0477-6 (PMC4522135; doi:10.1186/s12866-015-0477-6)
Supplement: Additional file 3: Figure S1. — Relative abundance of Haemophilus at 9 and 18 months of age in individuals with or without early life infections (A) and older siblings (B). [file 12866_2015_477_MOESM3_ESM.docx]

Figure S1 – Relative abundance of *Haemophilus* at 9 and 18 months of age in individuals with or without early life infections (A) and older siblings (B). Blue colored triangles indicate presence of early life infection or siblings, whereas red colored dots indicates no presence of these parameters. Statistical significance was evaluated by Mann Whitney test, using *p* < 0.05 as a measure of significance. *ns* = not significant.
