# Supplementary material for: Having older siblings is associated with gut microbiota development during early childhood
Source: BMC Microbiol. 2015 Aug 1;15:154. doi: 10.1186/s12866-015-0477-6 (PMC4522135; doi:10.1186/s12866-015-0477-6)
Supplement: Additional file 4: Figure S2. — Correlation matrices relating relative abundance of gut bacterial genera at 9 months (A) and 18 months (B) to the presence of asthmatic bronchitis and eczema. [file 12866_2015_477_MOESM4_ESM.docx]

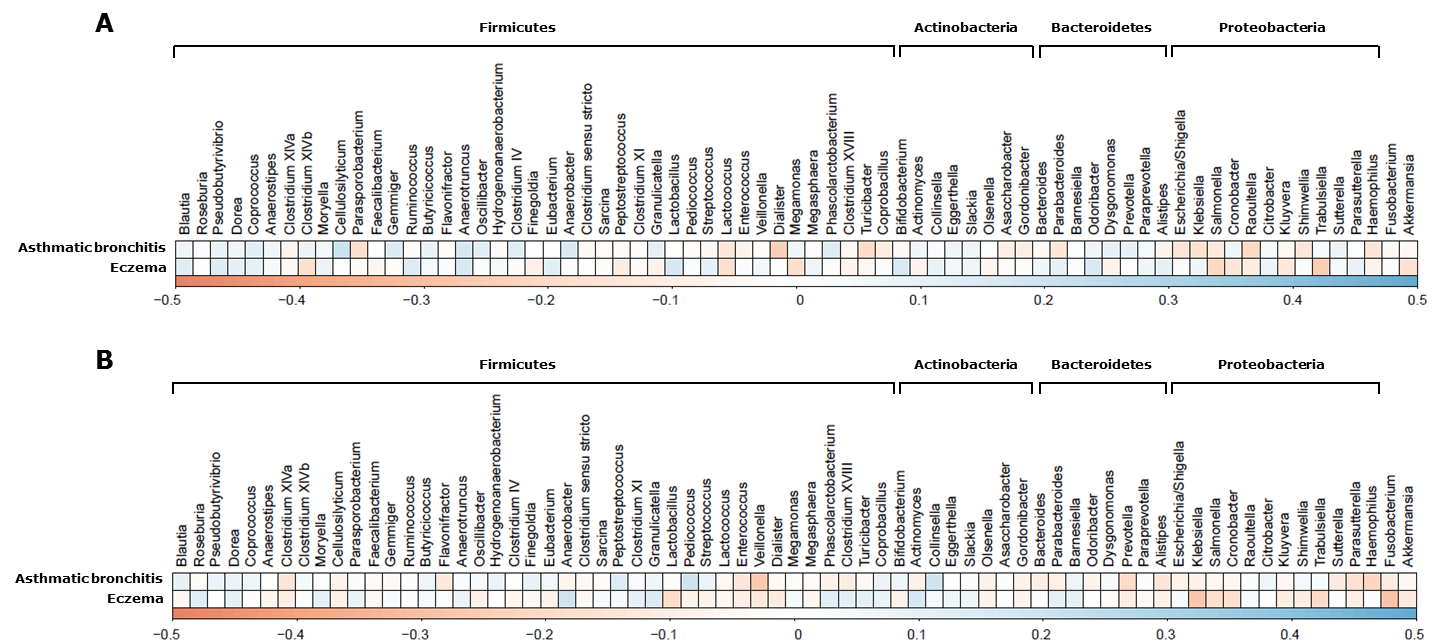


**Figure S2** – Correlation matrices relating relative abundance of gut bacterial genera at 9 months (A) and 18 months (B) to the presence of asthmatic bronchitis and eczema. Scale indicate the Spearman´s rank correlation coefficient rho, ranging from -0.5 (negative correlation; red color) to 0.5 (positive correlation; blue color).
