# Supplementary material for: Having older siblings is associated with gut microbiota development during early childhood
Source: BMC Microbiol. 2015 Aug 1;15:154. doi: 10.1186/s12866-015-0477-6 (PMC4522135; doi:10.1186/s12866-015-0477-6)
Supplement: Additional file 5: Figure S3. — Boxplots comparing bacterial diversity and richness at 9 and 18 months between individuals with or without eczema (A-B) and asthmatic bronchitis (C-D). [file 12866_2015_477_MOESM5_ESM.docx]

Figure S3 – Boxplots comparing bacterial diversity and richness at 9 and 18 months between individuals with or without eczema (A-B) and asthmatic bronchitis (C-D). White color indicates bacterial diversity or richness measured at 9 months of age, whereas grey color indicates bacterial diversity or relative richness measured at 18 months of age. Boxes show 25th to 75th percentiles and whiskers indicate minimum to maximum values. Statistical significance was evaluated by Mann Whitney test, using *p* < 0.05 as a measure of significance. *ns* = not significant.
